# A Numerical Investigation on Droplet Bag Breakup Behavior of Polymer Solution

**DOI:** 10.3390/polym12102172

**Published:** 2020-09-23

**Authors:** Guidong Chu, Lijuan Qian, Xiaokai Zhong, Chenlin Zhu, Zhongli Chen

**Affiliations:** College of Mechanical and Electrical Engineering, China Jiliang University, No. 258 Xueyuan Road, Hangzhou 310018, China; S1901081104@cjlu.edu.cn (G.C.); P1801085281@cjlu.edu.cn (X.Z.); zhuclgary@foxmail.com (C.Z.); chenzhongli@cjlu.edu.cn (Z.C.)

**Keywords:** polymer solution droplet, numerical simulation, droplet deformation, energy evolution, drag coefficient

## Abstract

The deformation and breakup of a polymer solution droplet plays a key role in inkjet printing technology, tablet-coating process, and other spray processes. In this study, the bag breakup behavior of the polymer droplet is investigated numerically. The simple coupled level set and volume of fluid (S-CLSVOF) method and the adaptive mesh refinement (AMR) technique are employed in the droplet breakup cases at different Weber numbers and Ohnesorge numbers. The nature of the polymer solution is handled using Herschel–Bulkley constitutive equations to describe the shear-thinning behavior. Breakup processes, external flow fields, deformation characteristics, energy evolutions, and drag coefficients are analyzed in detail. For the bag breakup of polymer droplets, the liquid bag will form an obvious reticular structure, which is very different from the breakup of a Newtonian fluid. It is found that when the aerodynamic force is dominant, the increase of the droplet viscous force will prolong the breakup time, but has little effect on the final kinetic energy of the droplet. Moreover, considering the large deformation of the droplet in the gas flow, a new formula with the cross-diameter (*D_cro_*) is introduced to modify the droplet drag coefficient.

## 1. Introduction

The fluids used in many spray processes mostly contain various kinds of additives, such as polymers and nanoparticles, which can make rather dramatic changes to the rheological behavior of the fluids. These fluids usually exhibit non-Newtonian behaviors, such as viscoelastic, shear-thickening, and shear-thinning [1]. The fluids formed by adding and mixing more than one type of nano-particles to the host liquid are called nanofluids, which can be used in fuel [2], atomizers [3], plasma studies [4], and heat energy exchangers [5]. The formation process of the polymer solution is similar to nanofluid. It is remarkable that polymer solutions are widely used in many spray processes due to their facile accessibility and easily adjust rheology. However, these nonlinear properties of polymer solutions make the droplet breakup process, which is a fundamental phenomenon in the spray processes, more complicated. Especially in some applications, for instance, inkjet printing technology in the textile industry [6], tablet coating process [7], the atomization of gelled kerosene in the ramjet [8], and the application of the inkjet etching of polymers to organic electronic devices [9]. The breakup of droplets has a great influence on determining spray characteristics, which further affects the mixture formation and surface quality. Therefore, a good knowledge of droplet breakup is significant for the development of spray processes.

To understand the droplet breakup process, experimental studies have been extensively conducted. It has been found that the droplet breakup of a Newtonian fluid is mainly governed by the Weber number (*We*) and Ohnesorge number (*Oh*). For a low Ohnesorge number (*Oh* < 0.1), with increasing the *We* number, the five breakup typical breakup modes occur: vibrational, bag, multimode, sheet-thinning, and catastrophic [1]. Among these various modes, our study focuses on bag breakup mode. One important reason is that this mode marks the onset of guaranteed breakup of the droplet from the standpoint of atomization. The properties of Newtonian fluids are relatively simple, and most of the existing studies focus on Newtonian droplets. Chou and Faeth [10] used the various Newtonian liquids to investigate the temporal properties of the bag breakup behavior. They found that the droplet will have a large acceleration due to the increase of the cross-stream diameter and the drag coefficient caused by droplet deformation. Zhao et al. [11] observed the Newtonian droplet bag breakup by using the high-speed camera. They studied the deformation of the droplet and proposed a theoretical formula to predict the appearance of the bag breakup mode. Kulkarni and Sojka [12] measured the parameters connected to droplet deformation, including the thickness of the rim, bag thickness, and total radial extent. After being made dimensionless, the changes of these parameters are theoretically deduced and finally confirmed by experimental findings. Recently, Zhao et al. [13] experimentally investigated the effect of turbulence on droplet breakup and observed the bag structure increases with the rise of the turbulence intensity. And they found that turbulence has a significant effect on the formation of the liquid bag structure. In general, the studies on the bag breakup behavior of Newtonian fluids are abundant. However, compared with Newtonian fluids, the experimental studies on non-Newtonian fluid droplet breakup is limited. Most were performed to determine the effects of viscoelastic and pure viscous properties on the droplet breakup [14,15,16]. Additionally, studies involving bag breakup are rarer. Snyder et al. [17] investigated elastic non-Newtonian liquid droplets in detail by high-speed camera. It was found that Newtonian and inelastic non-Newtonian droplets are similar in the bag breakup modes, but with differences in the formation of the bag. Zhao et al. [18,19] paid attention to the influence of rheological properties on the coal-water slurry (CWS) droplet breakup and showed that the breakup process will occur in a transition mode: hole breakup. They also found that hole breakup is slightly alike to the bag breakup of the Newtonian droplet, the difference being that, in hole breakup, there is no thin bag structure. Therefore, for polymer solutions, the effect of its complex rheological properties on the droplet bag breakup behavior needs further research.

In addition to the experimental studies, some computational studies have been performed, which can provide more details of the breakup processes. Moreover, the numerical simulation can provide flow field and energy evolution which are difficult to obtain in the experiments. The numerical simulation methods mostly used are the interface capturing methods, such as level set (LS) [20], volume of fluid (VOF) [21], and the coupled level set and volume of fluid (CLSVOF) [22] methods. Among them, the CLSVOF method takes advantage of the mass conservation of the VOF method and the sharp interface capturing of the LS method. Albadawi et al. [23] further proposed a simple CLSVOF (S-CLSVOF) to improve the surface tension implementation. In this method, only the VOF advection equation is solved, the interface normal is calculated by the re-initialization equation, and the physical properties are updated from a smoothed Heaviside function. Many studies of droplet bag breakup with these methods have been reported in the literature. Jallal and Mehravaran [24] studied the bag breakup mode of a single falling droplet by using an adaptive VOF technique and analyzed the mechanism of the breakup in detail. Xiao et al. [25] investigated the deformation and breakup of the droplet in supersonic flow by using the CLSVOF method to capture the interface. At different Weber numbers, the droplet deformation and breakup morphology are well predicted. They also compared drag coefficient *C_D_* and cross-stream dimension *D_c_* of the droplet in subsonic and supersonic flows. Yang et al. [26,27] used the S-CLSVOF method to study the density ratio influence on the atomization and the transitions of breakup modes. Under the highly unstable conditions, the drag coefficient, liquid surface structure, and the morphology of droplet breakup were investigated. They also found that the increase of *Oh* numbers leads to an increasing insensitiveness of the transition behaviors to the *We* number. Jiao et al. [28] used direct numerical simulation (DNS) coupled with the VOF method to study the droplet deformation processes with various turbulent velocity profiles, and showed that the deformation of a droplet in turbulent fields presents turning, rotation, and squeezing characteristics. For the numerical simulation of droplet breakup, researchers mostly take Newtonian fluids as the object. Most of the research contents are of the breakup of droplets under different environmental conditions. However, there are relatively limited studies on the breakup process of non-Newtonian fluids in airflow. In particular, Tavangar et al. [29] used coupled VOF and large eddy simulation (LES) methods with an adaptive mesh refinement technique to study the dynamic behavior of coal–water slurry (CWS) droplets during breakup. It was found that numerical results are in good agreement with experimental results. As mentioned above, compared with the studies on the Newtonian liquid, the research on the non-Newtonian droplet breakup is mainly focused on the breakup morphology and some characteristic parameters in the breakup process. However, the research on the bag breakup of polymer solution droplets and the kinetic energy evolution of the droplets with complex rheological properties have not been fully carried out. Therefore, in this paper, a representative polymer solution is selected to investigate the droplet bag breakup behavior by numerical simulation and applied the simple coupled level set and volume of fluid (S-CLSVOF) method and the adaptive mesh refinement (AMR) technique to capture the gas-liquid interface. The external flow fields, droplet deformation, energy evolution, and drag coefficients during polymer solution droplet breakup are mainly analyzed in detail. It is expected that this study can provide a broader theoretical basis for the improvement of polymer solutions atomization technology, and provide guidance and suggestions for the design of atomization nozzles and the setting of airflow environmental conditions. Specifically, during the processing of spray combustion, the atomization effect of fuel determines the efficiency of combustion. This is based on the results of the droplet breakup.

The paper is structured as follows. In Section 2 the governing equations, the constitutive equations, and the computational domain setup are presented. Section 3 shows the verification, including verification of the grid independence and numerical model verification. Section 4 offers the results and discussion, wherein the bag breakup behaviors are discussed in detail. In Section 5 the conclusions are summarized.

## 2. Numerical Method 

### 2.1. Governing Equations

The problem considered in this work is the polymer solution droplet breakup in airflow. The simple coupled level set and volume of fluid (S-CLSVOF) method is used in our paper, which can accurately capture the complex process of the interface evolution and has good mass conservation. The S-CLSVOF multiphase model has been proven to be capable in revealing the deformation mechanisms of liquid droplets by Yang et al. [26]. In their work, they give a comparison of the trajectory for the bag breakup between experimental data and the predictions by both VOF and S-CLSVOF models. And found that the S-CLSVOF method is in a closer agreement with the experimental measurement. Yamamoto et al. [30] also examined the deformation of a liquid drop in a square gas region by both VOF and S-CLSVOF methods. And results show that the S-CLSVOF method has higher accuracy compared with VOF. In this paper, Liquid and gas phases are supposed to be isothermal, immiscible, and incompressible fluids. The governing equations, including the continuity, momentum, and phase-fraction equation, can be written as:

(1) ∇⋅U=0

(2) $$\frac{\partial \rho U}{\partial t}+\nabla \cdot \left(\rho UU\right)=-\nabla p+\nabla \cdot \tau +F_{\sigma }$$

(3) $$\frac{\partial \alpha }{\partial t}+\nabla \cdot \left(\alpha U\right)=0$$

where **U** is the velocity vector of the flow field, *p* is the pressure, **F***_σ_* is the volume force, and *ρ* is the fluid density. The phase fraction *α* is used to represent a space mesh cell whether is occupied by the dispersed phase or the continuous phase. When the cells are full of the dispersed phase, the value of *α* is unity; the continuous phase corresponds to zero. When the mesh cells contain both the dispersed phase and the continuous phase, the value of *α* is between 0 and 1, which denotes an interface between the two phases.

In the original VOF function (3), the additional compression term ∇ ⋅ (**U**_c_ *α*(1 − *α*)) is introduced to sharpen the interface [22]. The improved VOF equation can be written as:

(4) $$\frac{\partial \alpha }{\partial t}+\nabla \cdot \left(\alpha U\right)+\nabla \cdot \left[U_{c}\alpha \left(1-\alpha \right)\right]=0$$

where **U**_c_ is the compression velocity to suppress diffusion of the interface, which can be calculated as follows:

(5) $$U_{c}=min\left(c_{\alpha }\left|U\right|,\max\left(\left|U\right|\right)\right)\cdot \frac{\nabla \alpha }{\left|\nabla \alpha \right|}$$

where *c_α_* is compression coefficient, generally a constant greater or equal to 1. The term ∇*α*/|∇*α*| is introduced convection of volume fraction function normal to the interface. Then the term *α*(1-*α*) can be used to ensure itself invalid in the outside of the interfacial area and the divergence operating guarantees conservation in terms of the entire compression term. The phase-fraction transport equation for the VOF function *α* is solved in the whole computational domain as a guarantee of the mass conservative nature.

At the same time, introducing the level set function *ψ* to ensure the interface smoothness by calculating smoother curvature. The method initializes the LS field by using *ψ*_0_ = (2*α* − 1)Γ as prime *ψ* guess value, where Γ is a non-dimensional number. Then re-distanced by a reinitialized equation. The formula is as follows:

(6) $$\frac{\partial \psi }{\partial \tau }=S\left(\psi_{0}\right)\left(1-\left|\nabla \psi \right|\right)$$

where *τ* is the artificial time step. *S*(*ψ*_0_) is a sign function defined with *S*(*ψ*_0_) = *ψ*_0_*/|ψ_0_|*. The solution of *ψ* converges to |∇*ψ*| = 1, which is a signed distance function around the interface, and the interface position is defined at the contour-line *ψ* = 0. The number of iterations (*ψ_corr_*) meet the following condition:

(7) $$\psi_{corr}=\frac{\varepsilon }{\Delta \tau }$$

where *ε* is the interface thickness. Then the interface normal vector **n** obtained from the LS field function is also used to calculate the interface curvature *κ* and volume surface tension *F_σ_* to ensure the accuracy of the interface calculation.

(8) $$\kappa =\nabla \cdot n=\nabla \cdot \left(\frac{\nabla \psi }{\left|\nabla \psi \right|}\right)$$

(9) $$F_{\sigma }=\sigma \kappa \left(\psi \right)\delta \left(\psi \right)\nabla \psi$$

(10) $$\delta \left(\psi \right)=\{\begin{array}{ll} 0 & \left|\psi \right|>\varepsilon \\ \frac{1}{2\varepsilon }\left(1+\cos\left(\frac{\pi \psi }{\varepsilon }\right)\right) & \left|\psi \right|\leq \varepsilon \end{array}$$

where *σ* is the surface tension coefficient and *δ* is the Dirac function used to ensure that the surface tension acts on the gas-liquid interface. The physical properties and the fluxes across the cell faces can be defined using a smoothed Heaviside function:

(11) $$H\left(\psi \right)=\{\begin{array}{ll} 0 & \psi <-\varepsilon \\ \frac{1}{2}\left[1+\frac{\psi }{\varepsilon }+\frac{1}{\pi }\sin\left(\frac{\pi \psi }{\varepsilon }\right)\right] & \left|\psi \right|\leq \varepsilon \\ 1 & \psi >\varepsilon \end{array}$$

(12) $$\rho =H\rho_{l}+\left(1-H\right)\rho_{g}$$

(13) $$\mu =H\mu_{l}+\left(1-H\right)\mu_{g}$$

where *μ*_1_ and *ρ*_1_ are the viscosity and density of the liquid droplet, *ρ_g_* and *μ_g_* are the density and viscosity of the gas.

The study of droplet breakup is inseparable from some dimensionless parameters. Commonly used dimensionless numbers include Weber number (*We*), Ohnesorge number (*Oh*), Reynolds number (*Re*), and non-dimensional time (*T*). At higher flow velocity, inertial forces deform the drop and lead to its breakup, which is characterized by the aerodynamic Weber number. The Ohnesorge number relates the viscous forces to inertial and surface tension forces. The Reynolds number is used to describe the ratio of the inertial force to the viscous force. As the breakup process is not instantaneous, experimentally observed times are typically made non-dimensional using the characteristic time. The detailed definition is as follows:

(14) $$We=\frac{\rho_{g}U_{r}^{2}D}{\sigma }\;,\;Oh=\frac{\mu_{l}}{\sqrt{\rho_{l}D\sigma }}\;,\;Re=\frac{\rho_{g}U_{r}D}{\mu_{g}}\;,\;T=t\frac{U_{r}}{\varepsilon^{0.5}D}$$

where *U_r_* is the initial relative velocity between the droplet and ambient, σ is the surface tension, *t* is the dimensional time, *θ* is the drop-to-ambient density ratio, *D* is the initial diameter of polymer droplet.

### 2.2. The Constitutive Equations

In this paper, the non-Newtonian water/carbomer solutions are taken into account. Its shear-thinning property is approximated by the Herschel–Bulkley rheological model [31]. Herschel–Bulkley model is one of the basic non-Newtonian fluid rheological models. The constitutive equation of the Herschel–Bulkley model is commonly written as $\tau =\tau_{0}+\kappa {\dot{\gamma }}^{n}$, where *τ* is the shear stress,$\dot{\gamma }$ is the shear rate, *κ* is the consistency index, *τ*_0_ is the yield stress. *n* defines the shear-thinning (*n* < 1) or shear-thickening (*n* > 1) behavior. 

The model can be rewritten as a generalized Newtonian fluid model to define the apparent viscosity of polymer solution as [32,33],

(15) $$\mu_{eff}=\{\begin{array}{cc} \mu_{0} & \left|\dot{\gamma }\right|\leq {\dot{\gamma }}_{0}\\ k{\left|\dot{\gamma }\right|}^{n-1}+\tau_{0}{\left|\dot{\gamma }\right|}^{-1} & \left|\dot{\gamma }\right|\geq {\dot{\gamma }}_{0} \end{array}$$

where *μ_0_* is the zero shear viscosity.

For non-Newtonian fluids, considering the effects of viscous dissipation and elastic recovery, the *Oh* number changes as follows [1]:

(16) $$Oh_{eff}=\frac{k}{D^{n-1/2}U_{r}^{1-n}\sqrt{\rho_{l}\sigma }}$$

### 2.3. Computational Domain Setup

**Figure 1:**
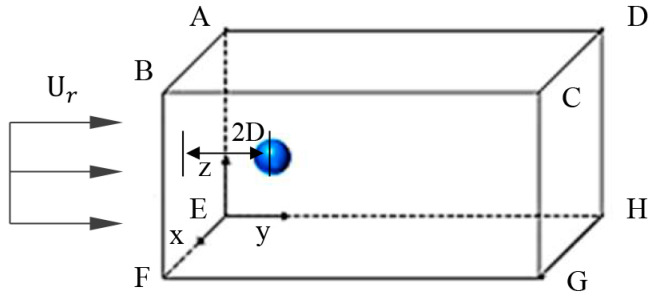
Schematic of the computational domain.

The computational domain is shown in Figure 1. A polymer solution droplet with a diameter of *D* = 5 mm is placed in the cube computational domain. The droplet is placed on the normal axis of the gas inlet boundary surface, and the distance from the boundary surface is controlled as 2*D*.

The setup of the computational domain needs to consider both the size and the grid resolution. A suitable computational domain size can ensure that the boundary conditions do not affect the simulation. There are four computational domain sizes for simulation verification. The computational domain size settings and the initial stage of the droplet are shown in Table 1.

**Figure 2:**
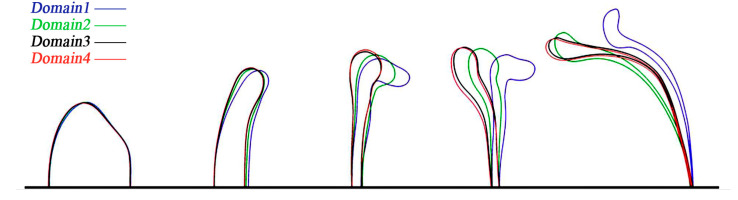
The evolutions of droplet morphology for various domain sizes.

**Figure 3:**
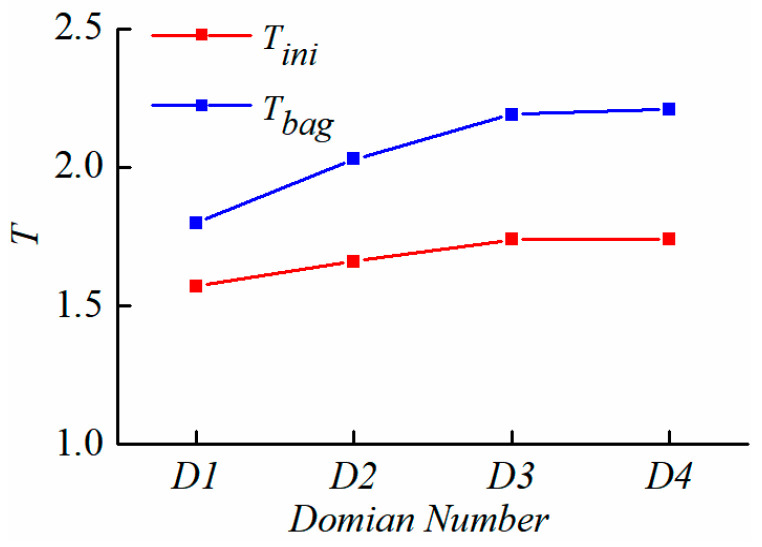
Comparison of droplet breakup time under various domain sizes.

The deformation and breakup of the droplet are simulated under different computational domain size. The results are as shown in Figure 2 and Figure 3. Figure 2 shows the evolution of droplet morphology in four domains and Figure 3 shows a comparison of the dimensionless time for different computational domain sizes.

As shown in Figure 2, in the four computational domains, the difference in the initial stage of droplet deformation is not obvious. Then, as the droplet deformation increases, the droplet morphology is significantly different between the small domain size and the larger one. While the computational domain size is larger than 8D · 8D · 24D (Domain 3), the difference of the droplet morphology will be reduced. In Figure 3, it is also found that after the computational domain reaches the Domain 3 (8D · 8D · 24D) domain, the initial breakup time *T_ini_* and the bag breakup time *T_bag_* no longer change obviously. The initial breakup time is defined as the interval required for a droplet to deform beyond the oblate spheroid shape, and the bag breakup time is defined as the time when the bag first breaks. Finally, the computational domain size 8D · 8D · 24D (Domain 3) is selected for simulation.

## 3. Verification

### 3.1. Grid Independency

**Figure 4:**
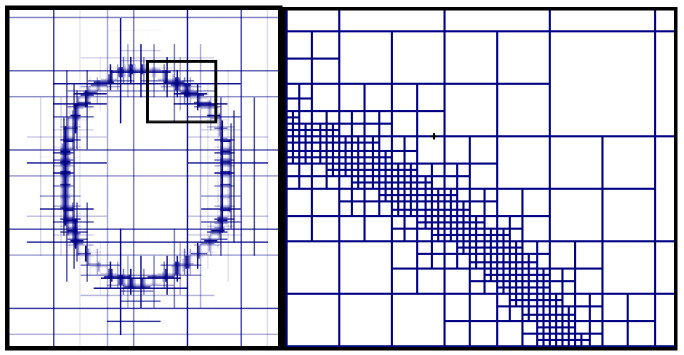
Adaptive grid on the interface.

The grid size of the interface, determining the accuracy and numerical stability of droplet deformation simulation, is important for capturing the liquid-gas interface. The result of using adaptive mesh refinement near the interface shows in Figure 4. Compared with the global refinement of the grid, it can greatly reduce the calculation time and storage cost.

**Figure 5:**
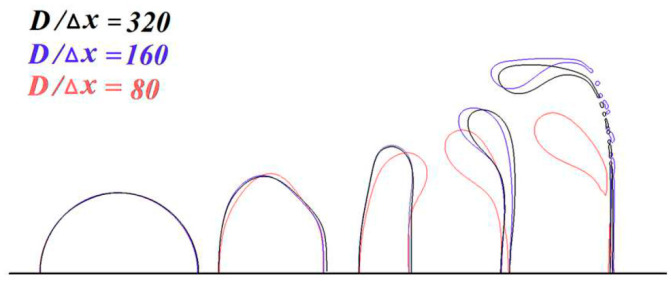
Resolution test with different grids.

The contours of the droplet related to three numerical resolutions with *D*/Δ*x* = 80, 160 and 320 are given in Figure 5, where Δ*x* is the minimum grid size. The results demonstrate that the droplet deformation and breakup are nearly independent of the mesh size when the *D*/Δ*x* is larger than 160. Therefore, the interface grid resolution of *D*/Δ*x* = 160 is adopted to research the deformation and the fragmentation of the droplet in all of the following simulations.

### 3.2. Numerical Model Verification

**Figure 6:**
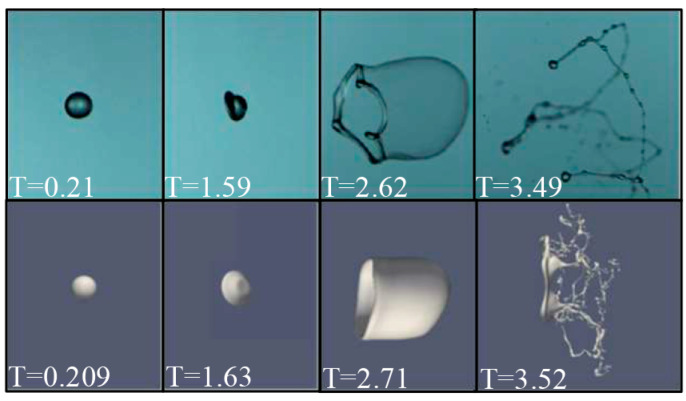
Comparison of the numerical results with experimental results for droplet bag breakup.

To validate the accuracy of the numerical model, the simulations are compared with the experimental data. In the experiment, the carbomer solution with a mass fraction of 0.015 wt% was chosen for the experimental study of droplet breakup. The single carbomer solution droplet injected into continuous air flows were investigated by using a high-speed camera under the condition of *We* = 22.09 and *Oh* = 0.0021. The comparison between the numerical and experimental results are shown in Figure 6. As can be seen, the numerical and experimental results are in good agreement. Therefore, the present numerical model, which is proven, can be used for the simulation of the non-Newtonian fluid droplet bag breakup.

## 4. Results and Discussion

In the present study, the influence of the *We* number and *Oh* number on the droplet breakup process is analyzed. The *Oh* number can be calculated by Equation (15). For the polymer solution (Carbomer), the liquid density *ρ_1_* is 1000 kg/m^3^, and the surface tension *σ* is 0.0751 kg/s^2^. The computational parameter settings of the numerical simulation cases are shown in Table 2.

### 4.1. Bag Breakup Behavior and Velocity Field

**Figure 7:**
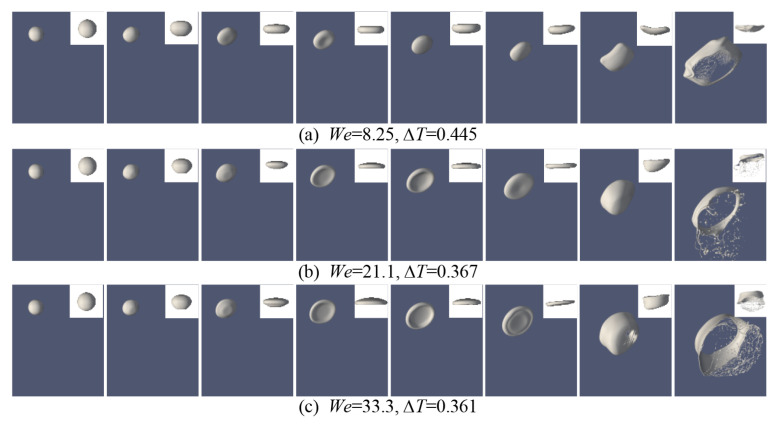
Bag breakup behavior of droplet under different *We* numbers (*Oh* = 0.002). (**a**) *We* = 8.25, Δ*T* = 0.445; (**b**) *We* = 21.1, Δ*T* = 0.367; (**c**) *We* = 33.3, Δ*T* = 0.361.

The process of bag breakup can be divided into four stages: deformation, bag growth, bag breakup, and ring breakup [10]. For comparison, the simulations with *We* of 8.25, 21.1, and 33.3 are respectively implemented to study the effect of *We* on the droplet breakup morphology. A sequential picture of droplet deformation and breakup is shown in Figure 7. In the stage of deformation, for the case of *We* = 8.25, the thickness of the oblate droplet is larger than in other cases. Additionally, the breakup of the droplet is not a bag breakup in this case, but close to that. As the *We* number increases to 33.3, the thickness of the droplet becomes thinner during the deformation. The droplets can form an obvious bag shape under the action of airflow. The droplet deformation degrees have expanded significantly. However, compared with the experiment results from Kulkarni et al. [12], the fragmentation phenomenon is different. In their experimental cases, both the bag rim and the bag break into small droplets. In this numerical case, the non-Newtonian droplet has a mostly intact rim, and the bag forms ligaments, not droplets, as it breaks up. The bag looks like a reticular morphology.

**Figure 8:**
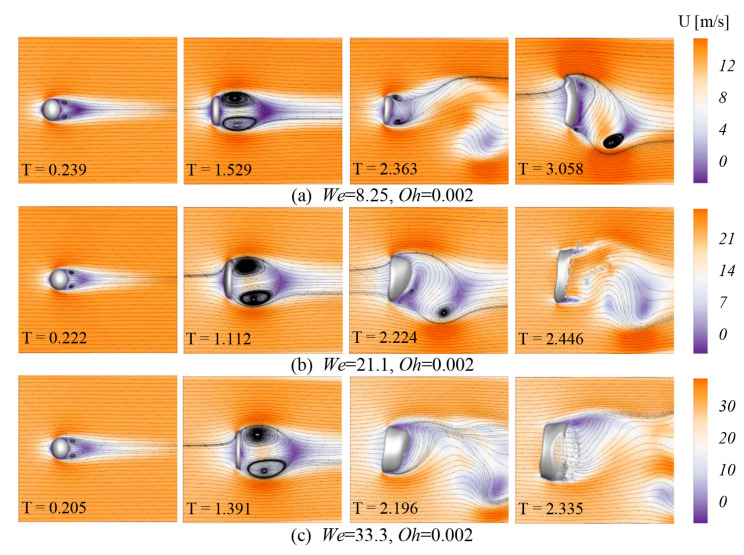
Velocity field under different *We* numbers (*Oh* = 0.002). (**a**) *We* = 8.25; (**b**) *We* = 21.1; (**c**) *We* = 33.3.

The deformation of a droplet is closely related to the velocity field around it. For all cases in Figure 8, since ambient air flows around the droplet, a symmetric vortex pair appears behind the drop in the initial stage (T ≈ 0.2), and the velocity is slower in this region. Over time, the size of these two vortices is increasing. This non-uniform velocity distribution finally results in the oblate droplet. Then, the center of the droplet gets blown downstream and the bag start to extend. The vortices begin to gradually show asymmetry under the influence of the bag growth and separate from the back of the liquid bag. It is worth noting that vortices also affect droplet shape. As shown in Figure 8b, with the influence of the symmetry vortices, the droplet will appear “inverted bowl” shape at the breakup time T = 1.112 (Figure 8b). However, the droplet, which is affected by the vortices, can still form a bag shape. It can be found that the droplet deformation is affected by the airflow. After the drop deforms, it will affect the velocity distribution, which further leads to the occurrence of droplet breakup.

### 4.2. Droplet Deformation

**Figure 9:**
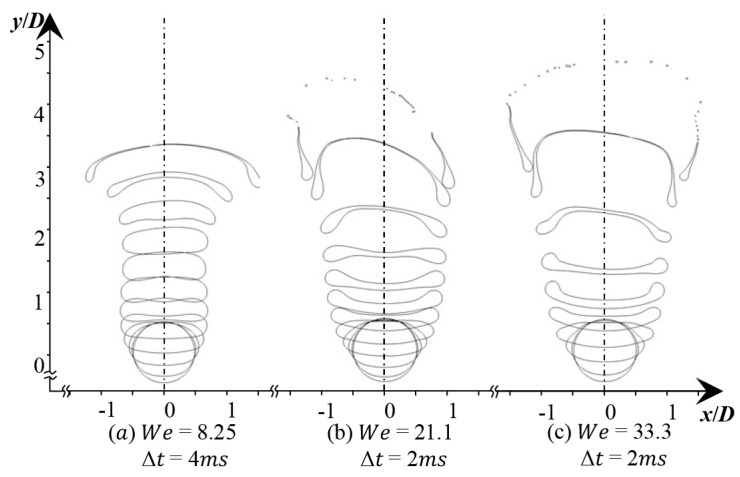
Evolution of droplet shape under different *We* numbers (*Oh* = 0.002).

The results obtained for the droplet shapes in the 2D domain are presented in Figure 9. Compared with Figure 7, the effect of the *We* number on the bag breakup is more clearly reflected. With the increase of the *We* number, the thickness of the droplet during the deformation stage gradually decreases, and the droplet becomes more easily break. But during the breakup, the liquid bag stretches into a filament, and only a small number of sub-droplets are separated from the mother droplet.

**Figure 10:**
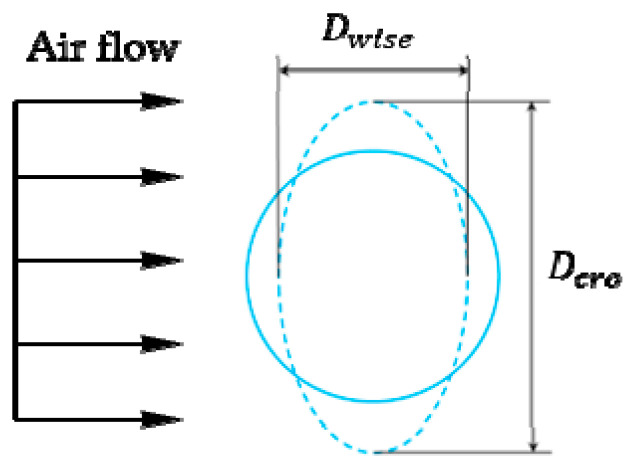
The deformation parameter of droplet.

The initial stage of droplet breakup is from spherical shape deform into a shape that can be approximated as an oblate ellipsoid. This is illustrated in Figure 10. Here *D_wise_* is the deformed drop diameter in the stream-wise direction and *D_cro_* is the deformed drop diameter in the cross-stream direction. These two diameters are converted into a dimensionless number by *D_wise_*/*D* and *D_cro_*/*D* to describe the degree of the droplet deformation.

**Figure 11:**
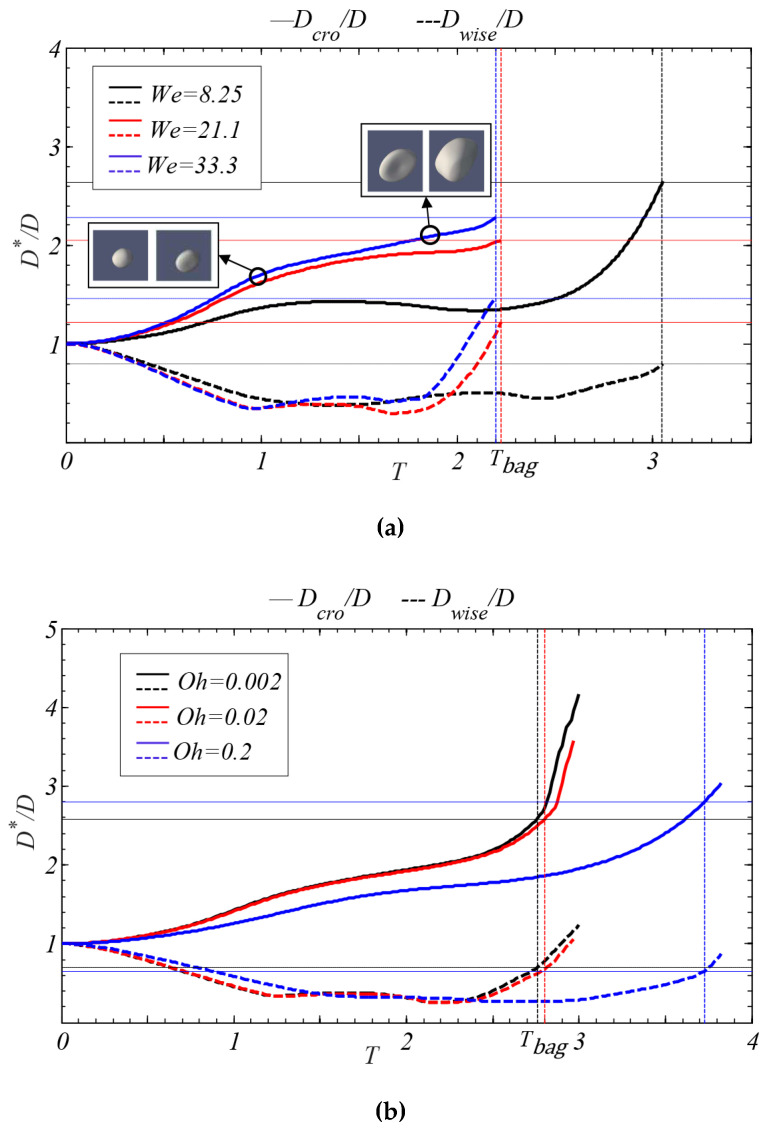
The temporal evolution of droplet characteristic diameter. (**a**) The droplet deformation under different *We* number (*Oh* = 0.002); (**b**) the droplet deformation under different *Oh* number (*We* = 18.5).

As shown in Figure 11, the temporal evolution of the droplet deformation is presented for three *We* numbers (8.25, 21.1, and 33.3) and three *Oh* numbers (0.002, 0.02, and 0.2). In Figure 11a, for the case of *Oh* = 0.002, the stream-wise deformation (*D_wise_*/*D*) initially decreases due to droplet flattening, followed by an increase owed to the formation of the liquid bag, while the cross-stream deformation (*D_cro_*/*D*) increases during the whole duration of the process. For the cross-stream deformation, although the trend shows an upward tendency, there are differences in each stage. In the initial stage, the cross-stream deformation (*D_cro_*/*D*) of the droplet will increase to a relatively fixed value. In the intermediate stage, the deformation will still increase, but the rate of rising will decrease. This indicates that there is a deformation block point between the initial and intermediate stages. As seen in Figure 11a, this point occurs at the droplet deformation transitions from an ellipsoid shape to a flat shape. After this point, the droplet will maintain a flat shape for a while. During this time, the energy which the droplet obtains from the aerodynamic force will be mainly used for the drop movement along the stream-wise direction of the airflow rather than deformation. In the final stage, the cross-stream deformation will increase sharply owed to the formation of the liquid bag. Similarly, there exists a deformation turning point at the beginning of the final stage, and this point appears as the droplet deformation transitions from a flat shape to a bag shape. For the effect of aerodynamic force on the breakup process, the increase of that results in a higher deformation rate. It is worth noting that the trend of droplet deformation is extraordinarily close at *We* = 21.1 and 33.3. This is mainly because the bag structure formed by the external aerodynamic force is in a relatively stable stage under these two conditions. At this stage, the deformation degree of the droplet is relatively stable.

In Figure 11b, as the *Oh* number increases, the tendency of droplet deformation decreases, which shows the opposite trend compared with Figure 11a. The mechanism of the droplet deformation and breakup is essentially determined by the interaction of aerodynamic and internal forces. The increase of *Oh* number presents the increase of viscous force which results in a lower deformation rate. When the *Oh* number is low (*Oh* = 0.02 and 0.002), the trend of droplet deformation has no significant difference. This is related to the viscous force of the droplet in these two cases. In these conditions, the dominant effect of droplet deformation is still the aerodynamic force, while the effect of the viscous force is small. Therefore, when the *Oh* value is low, the deformation degree of the droplet in the breakup process changes little. However, when *Oh* = 0.2 (*Oh* > 0.1), the droplet deformation is evidently inhibited.

### 4.3. Energy Evolution

**Figure 12:**
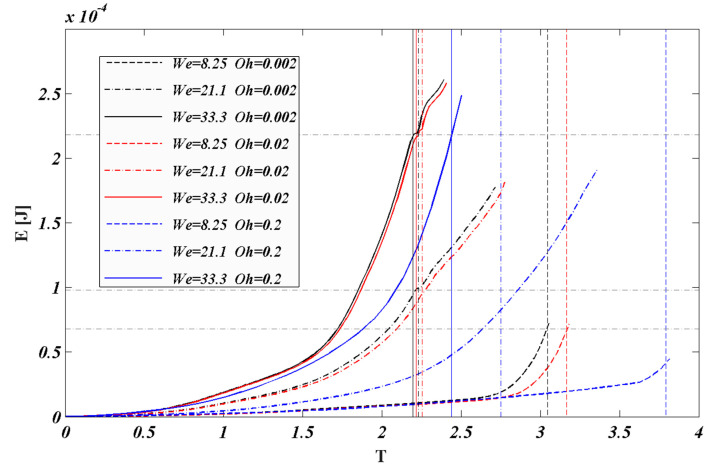
The dimensionless time evolution of droplet kinetic energy.

As shown in Figure 12, nine cases are selected for numerical studies to analyze the kinetic energy during the droplet breakup process. Based on the conservation of energy, the kinetic energy (*E*) of the droplet can be calculated as follows:

(17) $$E=\frac{1}{2}\sum_{i,j,k=1}^{N_{cells}}\left(\alpha_{cell}\nu_{cell}\rho_{l}\right)\left(u_{ijk}^{2}+v_{ijk}^{2}+w_{ijk}^{2}\right)$$

where *N_cells_* is the number of the computational cell. *α_cell_* is the volume fraction of the liquid phase. *v_cell_* is the volume of a computational cell. *ρ_1_* is the liquid density, *u_ijk_*, *v_ijk_* and *w_ijk_* are the velocity component. 

In the process of droplet breakup, the energy is provided by aerodynamic force. As shown in Figure 12, it is found that in the low *We* number (especially *We* = 8.25), the kinetic energy of the droplet is low when breakup. With the increase of the *We* number, the total kinetic energy of the droplet also raises. When the breakup occurs at the cases of the same *We* number, the increase in the viscous force has a hindering effect on the energy growth rate during the droplet movement, but this effect is clearly reflected when *Oh* = 0.2 (*Oh* > 0.1). Although the high viscous force will hinder the growth rate of the kinetic energy of droplets, the total kinetic energy of the droplet during the breakup process is approximate. Especially, for the cases of *We* = 33.3, the total kinetic energy of the droplet almost identical, and the kinetic energy value is 0.00022 J. However, the breakup time will be extended with the increasing of *Oh* number. The change of kinetic energy of droplet is closely related to the deformation of droplets. As shown in Figure 11b, at the same *We* number, the deformation degree of the droplet shows no obvious difference at the breakup time, which is consistent with the trend of the droplet kinetic energy. Aerodynamic forces affect the deformation of droplets. In the process of droplet shape changing from spherical to flat, the contact area between droplet and airflow is determined by the cross-stream deformation of droplet. The faster the cross-stream deformation of the droplet changes, the larger the contact area between the airflow and the droplet surface. Eventually, the kinetic energy of droplet increases faster. According to the result of the analysis of droplet deformation, when the effect of aerodynamic force is higher, the viscosity of the droplet has less influence on the final evolution of kinetic energy. This leads to a situation that the kinetic energy of the droplet increases faster, but the final total kinetic energy is similar.

### 4.4. Drag Coefficient

The drag coefficient of the droplet is important for evaluating the process of droplet breakup. When a droplet is in ambient airflow, it will be affected by the airflow. The unequal distribution of the static pressure is formed over the droplet surface, which can cause the deformation of the droplet. As a result, the droplet will be compressed in the stream-wise direction of the airflow. In this process, the drag coefficient of the droplet will change greatly. 

According to Temkin and Kim [34], the standard drag coefficient for the droplet is:

(18) $$\frac{4}{3}\pi {\left(\frac{D}{2}\right)}^{3}\rho_{l}\frac{dU_{Droplet}}{dt}=\frac{1}{2}\rho_{g}{\left(U_{g}-U_{Droplet}\right)}^{2}C_{d}\frac{\pi D^{2}}{4}$$

In the definition of drag coefficient used here, considering that the droplet will deform in the airflow, the cross-stream diameter (*D_cro_*) is used in calculating the frontal area. Equation (18) can be transformed into:

(19) $$\frac{4}{3}\pi {\left(\frac{D}{2}\right)}^{3}\rho_{l}\frac{dU_{Droplet}}{dt}=\frac{1}{2}\rho_{g}{\left(U_{g}-U_{Droplet}\right)}^{2}C_{d}\frac{\pi D_{cro}^{2}}{4}$$

Then the droplet centroid velocity *U_Droplet_* and the drag coefficient *C_d_* can be calculated according to the following formula:

(20) $$U_{Droplet}=\frac{\sum_{\alpha >0.5}U_{cell}\cdot v_{cell}}{\sum_{\alpha >0.5}v_{cell}}$$

(21) $$C_{d}=\frac{4}{3{\left(U_{g}-U_{droplet}\right)}^{2}}\frac{D^{3}}{D_{cro}^{2}}\frac{dU_{Droplet}}{dt}\frac{\rho_{l}}{\rho_{g}}$$

where *U_g_* is the velocity of the air, *v_cell_* is the dimensional volume of a single grid, *U_cell_* is the computational grid speed.

**Figure 13:**
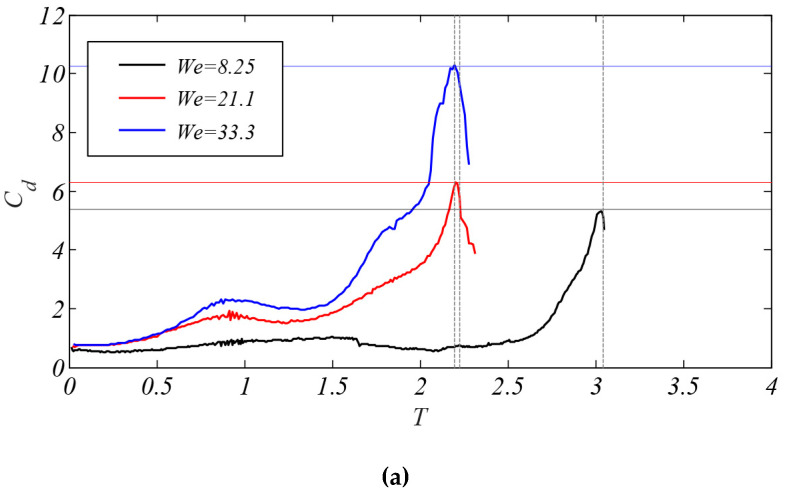

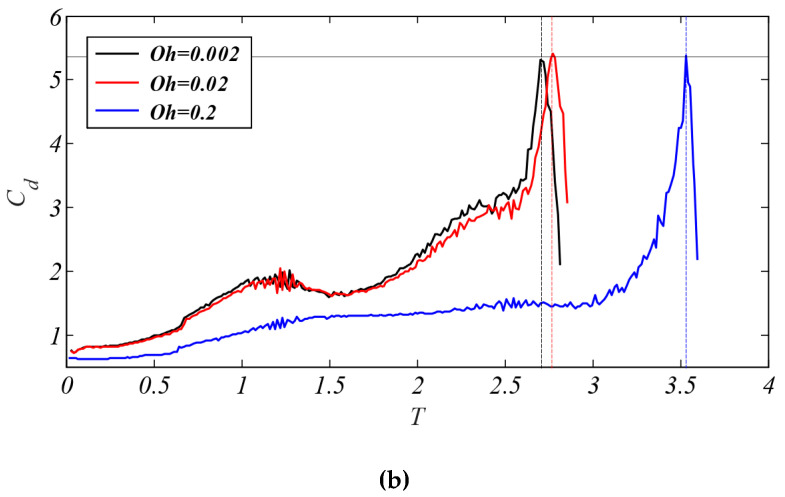
The temporal evolution of the droplet drag coefficient. (**a**) The drag coefficient under different *We* number (*Oh* = 0.002); (**b**) the drag coefficient under different *Oh* number (*We* = 18.5).

Figure 13 shows the evolutions of the droplet drag coefficient under different *We* (*We* = 8.25, 21.1, and 33.3) and *Oh* (*Oh* = 0.002, 0.02, and 0.2) numbers. As shown in Figure 13a, as the *We* number increases, the drag coefficient raises, which is related to the kinetic energy of the droplet. The growth of kinetic energy can accelerate the deformation of the droplet. The deformation in the cross-stream direction causes an enlargement for the contact surface between the droplet and the airflow. Then the resistance force on the leeward side of the droplet amplifies, resulting in a raise for the drag coefficient. According to the trend of kinetic energy in Figure 12, the energy growth rate will be faster with aerodynamic forces reinforced. Therefore, with the increase of the *We* number, the droplet drag coefficient not only continues to rise, but also rises faster. It is worth noting that the drag coefficient reaches its maximum value before the droplet breakup. After the bag breakup time, the drag coefficient decreases rapidly.

As shown in Figure 13b, the trend of droplet drag coefficient under different *Oh* numbers is the same as that in Figure 13a. However, when the bag breaks, the drag coefficient is close under the three cases (*Oh* = 0.002, 0.02, and 0.2). For the cases of *Oh* = 0.002 and 0.02, the evolution of the drag coefficient is basically in coincidence. According to the evolution of the kinetic energy, the increase of viscous force will have a slight effect on the droplet’s energy. As a result, the deformation of the droplet is similar when it is broken, causing the drag coefficient is so close. Additionally, with the *Oh* number increases, the growth trend of the droplet drag coefficient will slow down.

For the deformation of the bag breakup initial stage, it was found that *C_d_* largely was a function of the degree of deformation of the droplet from the research of Hsiang and Faeth [35]. Then Liu et al. [36] proposed the following empirical expression for the drag coefficient during droplet deformation:

(22) $$C_{d}=C_{d,sphere}\left(1+2.632y\right)$$

where *y* represents the non-dimensional displacement of the drop equator, which can be written as *y* = 1 − (*D*/*D_cro_*)^2^. *C_d,sphere_* is the coefficient of drag for a sphere at the same Reynolds number *Re* > 2000. Its value is 0.47 here. 

**Figure 14:**
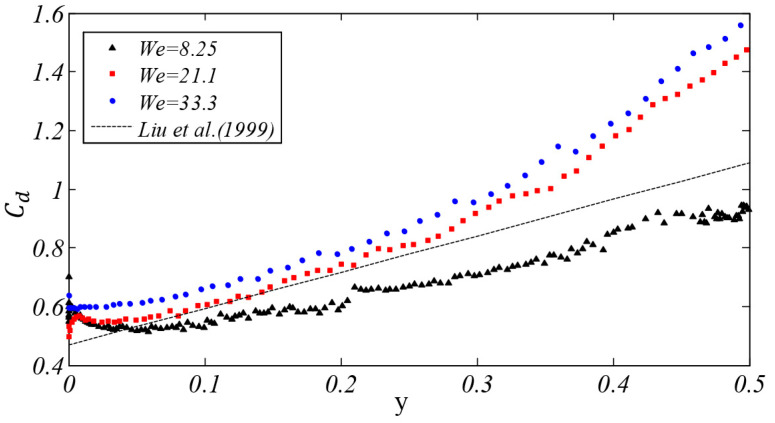
The relationship between the droplet equator displacement *y* and the drag coefficient.

As shown in Figure 14, the drag coefficient in the stage of droplet deformation under three cases (*We* = 8.25, 21.1, and 33.3) is compared with the result of Liu’s empirical expression. When *We* = 8.25, the drag coefficient obtained by simulation is lower than the result of the empirical expression. When the *We* number is higher (*We* = 21.1, 33.3), the drag coefficient which changes with the non-dimensional displacement is higher than the result of the expression. As the *We* number increases, the result shows that the drag coefficient in the deformation state will increase. It can be found that the empirical formula of Liu et al. is not suitable for describing the drag coefficient in this research.

## 5. Conclusions

Based on the simple coupled level set and volume of fluid method (S-CLSVOF) and adaptive mesh refinement (AMR) technique, the bag breakup of the polymer solution droplet was studied by numerical simulation. The S-CLSVOF method combines the advantages of VOF and LS methods and the AMR technique significantly reduces the computational cost. From numerical simulations, the effects of the velocity field on the breakup results of the polymer droplet were investigated. Furthermore, the droplet deformation, the energy evolution, and the drag coefficient were discussed in detail. The conclusions are as follows:

(1) The shear-thinning droplet bag breakup mode is similar to the Newtonian, but the difference is that the shear-thinning droplet has a mostly intact rim, and the liquid bag forms ligaments. Moreover, during the process of droplet breakup, the velocity distribution and the droplet deformation influence each other. The vortices located on the leeward side of the droplet changes from symmetrical to asymmetrical, which continuously affects the morphological change of the droplet.

(2) For polymer solution droplet bag breakup, when the Weber number is higher (*We* = 33.3), the increase of viscous force has little effect on the total kinetic energy of the droplet when it breaks. However, it will extend the breakup time significantly.

(3) Considering the deformation of the polymer droplet, the cross-stream diameter (*D_cro_*) is introduced to calculate the frontal area, the new formula of the drag coefficient is proposed. This formula can be used to estimate the transient drag during the bag breakup process. For the cases of moderate Weber number (*We* = 18.5) in the bag mode, the value of the drag coefficient is approximate when the droplet breaks up, which is not affected by the effect viscous force to some extent. 

## Figures and Tables

**Table 1:** Computational domain and initial condition setting.

Computational Domain	Computational Domain Size	Initial Variable	Initial Value
Domain 1	3D · 3D · 24D	*D* (mm)	5
Domain 2	4D · 4D · 24D	*We*	25
Domain 3	8D · 8D · 24D	*Oh*	0.001
Domain 4	16D · 16D · 24D	*ρ_1_*/*ρ_g_*	820
		*μ_1_*/*μ_g_*	56

**Table 2:** Parameter settings in simulation cases.

Case	*We*	*Oh_eff_*	Case	*We*	*Oh_eff_*	Case	*We*	*Oh_eff_*	Case	*We*	*Oh_eff_*
(a)	8.25	0.002	(d)	8.25	0.02	(g)	8.25	0.2	(j)	18.5	0.002
(b)	21.1		(e)	21.1		(h)	21.1		(k)		0.02
(c)	33.3		(f)	33.3		(i)	33.3		(l)		0.2

## Abbreviations/Nomenclature

*C_d_*	Drag coefficient	$\mu_{g}$	Air velocity
*D*	The initial diameter of liquid droplet	$\mu_{l}$	Liquid velocity
*D_cro_*	The cross-stream diameter	**U**	The velocity vector of the flow field
*D_wise_*	The stream-wise diameter	$U_{g}$	The velocity of the air
*E*	The kinetic energy	$U_{r}$	The initial relative velocity
$F_{\sigma }$	Volume surface tension	$U_{droplet}$	The droplet centroid velocity
k	The consistency coefficient	$U_{cell}$	The computational grid speed
*n*	The rheological index	$v_{cell}$	The volume of a computational cell
$N_{cell}$	The number of the computational cell	*We*	Weber number
*Oh*	Ohnesorge number	$\rho_{l}$	Liquid density
*Oh_eff_*	Effective Ohnesorge number	$\rho_{g}$	Air density
*p*	Pressure	σ	Surface tension
*Re*	Reynolds number	$\tau_{0}$	The yield stress
*T*	Non-dimensional time	$\dot{\gamma }$	Shear rate
*t*	Dimensional time	α	The phase fraction
*T_ini_*	Initial breakup time	$\alpha_{cell}$	The volume fraction of the liquid phase
*T_bag_*	Bag breakup time	$u_{ijk},v_{ijk},w_{ijk}$	The velocity component
$\mu_{0}$	Zero shear viscosity	ε	The interface thickness
$\mu_{eff}$	Apparent viscosity	θ	The drop-to-ambient density ratio
